# Polarity-Dependent EDM-Type Degradation in Rolling Bearings Under Low-Speed Unipolar Excitation

**DOI:** 10.3390/ma19112248

**Published:** 2026-05-26

**Authors:** Zifan Li, Ran Cai, Tianyi Zhang, Xueyuan Nie

**Affiliations:** Department of Mechanical, Automotive & Materials Engineering, University of Windsor, 401 Sunset Avenue, Windsor, ON N9B 3P4, Canada

**Keywords:** rolling element bearings, electrical discharge damage, stray shaft current, unipolar pulsed DC excitation, polarity-dependent degradation

## Abstract

Bearings in electric motors are exposed to stray currents and shaft voltages, which can accelerate surface damage and reduce service life. This study examines how pulsed direct current (DC) direction affects early-stage degradation in rolling bearings under low-speed operation. A dedicated test rig was used in which the bearing inner and outer rings were connected directly to the positive and negative terminals of a pulsed DC power supply. Unipolar excitation was applied at 20 kHz with a nominal current of 3 A and shaft-voltage peaks of about 3 V for 3 h, with current flowing in only one direction during each test. After testing, the bearings were sectioned and examined by optical microscopy, scanning electron microscopy (SEM), and X-ray photoelectron spectroscopy (XPS). The results showed that when current flowed from the outer ring to the inner ring, visible electrical discharge machining (EDM)-type damage was mainly found on the outer raceway. When the current direction was reversed, the damaged region shifted to the inner raceway. The affected areas showed crater-like discharge features and surface chemical changes, while the opposite raceway showed much less change under the same test conditions. These observations indicate that current direction influences where EDM-type damage more likely forms in the bearing under the present low-speed unipolar excitation conditions.

## 1. Introduction

Rolling element bearings operating in electromechanical systems are exposed to unintended electrical stress. Stray currents passing through bearing contacts are often linked to electrical discharge machining (EDM)-type damaging processes [1,2]. These processes can damage the raceway surface and lead to fluting, pitting, and local melting [3,4]. Early studies on electrically induced bearing damage often used simplified single-contact setups, including ball-on-disc test rigs, to examine how electrical discharge occurs under controlled lubrication conditions [5,6]. Component-level bearing test rigs were subsequently used to examine damage under more representative contact geometry and loading conditions [7]. A few review papers related to electrical bearing damage across nano- to micro-scales have provided an overview of the state of the art of research regarding effects of electrical bearing currents on damage of bearing components [8,9].

Those Studies move from simplified contact tests to bench tests that more closely reflect real operating systems. Liu et al. [10] tested an electric vehicle transmission under realistic driving profiles and compared the shaft-voltage states with discharge behavior and raceway damage. The results showed changes among resistive, mixed, and capacitive states as the lubricant film developed. Surface analyses after testing, including white-light interferometry (WLI), X-ray photoelectron spectroscopy (XPS), and focused ion beam/energy-dispersive X-ray spectroscopy (FIB/EDS), further elucidated material removal, microstructural changes, and oxide formation resulting from repeated discharge events [11]. Other studies have also examined how electrical and mechanical parameters affect discharge severity. Janik et al. [12] evaluated grease-lubricated rolling contacts across a broad voltage and frequency range, incorporating hybrid ceramic elements to assess the interruption of conductive pathways. Huan et al. [13] employed an orthogonal experimental design to quantify the coupled effects of voltage amplitude, excitation frequency, and mechanical load on the progression of electrical corrosion. Peng et al. [14] provided a comprehensive review of wind turbine bearing failures, identifying electrical erosion as a major contributor to long-term reliability degradation. At reduced rotational speeds, discharge activity can become localized and intermittent, making repeatable testing difficult and weakening the correlation between electrical signals and surface damage [15]. Most existing studies have focused on bipolar or sinusoidal excitation [16,17,18]. Because the current alternates across the bearing contact in these tests, the effect of current direction is difficult to separate.

In our previous study [19], the low-speed test rig was used with pulsed excitation to identify the voltage range for EDM-type bearing damage. The results showed that higher peak shaft voltage and current increased the discharge frequency and led to more surface damage. That study used a single connection polarity, thereby leaving unresolved the question of whether the current direction influences the distribution of damage between the inner and outer raceways. Rolling element contact fails studies under direct current (DC) have shown that polarity can affect the damage of material microstructures [20]. However, it is still unclear how this polarity effect influences EDM-type surface damage under low-speed unipolar pulsed excitation. Compared with sinusoidal or bipolar excitation [19], unipolar excitation has received less attention, where the unipolar excitation produces pulsed voltage and current in one direction. This directional characteristic has not been known if the electron flow through the rolling contact from shaft-to-housing or housing-to-shaft may alter discharge localization patterns and energy concentration.

This study examines how excitation polarity affects the early-stage degradation of rolling element bearings under controlled low-speed operation. For this test, the positive terminal was connected to the outer ring and the negative terminal to the inner ring, allowing current to pass through the rolling elements. The different power supply settings, unipolar positive and unipolar negative, were applied under identical waveform parameters (20 kHz, 3 A, 3 h). After testing, the raceway surfaces were examined by SEM, EDS, and XPS. The analysis focused on surface morphology, chemical composition, and whether the EDM-like damage showed any polarity-dependent difference.

## 2. Methods

All experiments were performed on a bearing test platform previously developed for low-speed electrical discharge investigations [19]. The experimental procedure combined visual inspection and electrical waveform monitoring.

### 2.1. Experimental Setup

The test bearing was a FAG deep-groove ball bearing made of SAE 52100 chrome steel. The bearing was lubricated with SKF LGHP 2 grease (AB SKF, Yokohama, Japan), which is a mineral-oil-based, polyurea-thickened grease commonly used for electric motor bearings. Before each test, approximately 1 mL of fresh grease was applied, corresponding to about 15% of the raceway volume.

**Figure 1:**
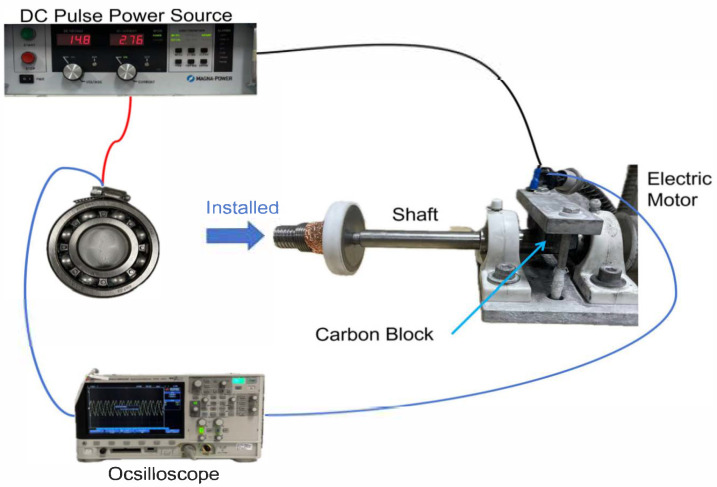
Experimental setup used for the low-speed unipolar excitation tests [19].

The mechanical assembly (Figure 1) consisted of a horizontal shaft supported by electrically insulated bearing housings to avoid unintended grounding paths. The test bearing was mounted at one end of the shaft, and the other end was driven through a belt wheel to maintain a constant rotational speed of 30 rpm. No external radial or axial load was intentionally applied during the tests, and no preload was used. A carbon block connector was used to stabilize the shaft-side electrical connection and to provide one measurement point for the oscilloscope. The opposite oscilloscope connection was made to the bearing outer ring. The excitation polarity was controlled by directly connecting insulated copper leads from the pulsed DC power supply to the exposed bearing ring surfaces. Under the unipolar-positive condition (Experiment 1), the positive terminal was connected to the outer ring and the negative terminal to the inner ring through the shaft and carbon connecting block. Under the unipolar-negative condition (Experiment 2), the polarity was reversed. This arrangement established a defined current path through the rolling contacts between the inner and outer raceways.

### 2.2. Electrical Monitoring and Control

**Figure 2:**
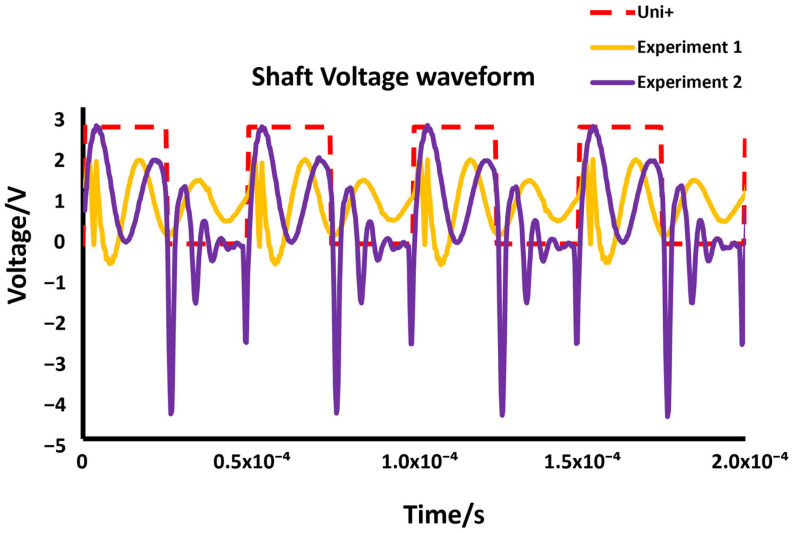
Power supply setting and average voltage waveform at 20 kHz.

The applied voltage waveform during each test was monitored using a Keysight InfiniiVision 3000T X-Series oscilloscope (Keysight Technologies, Inc, Bayan Lepas, Malaysia). The oscilloscope was connected between the carbon-block/shaft side and the outer-ring side to track the voltage response across the bearing during excitation. The waveform shown in Figure 2 represents the typical voltage response obtained under each test condition. Waveform stability was checked before and after each run to confirm that the imposed excitation remained consistent. Because rotational speed, lubrication condition, test duration, and electrical parameters were kept the same in both polarity conditions, the comparison focused on the effect of current direction under otherwise identical operating conditions.

### 2.3. Test Procedure

Before each test, the bearing exterior was cleaned with isopropanol to remove contaminants, and 1 mL of fresh grease was applied under the same lubrication condition for all trials. After installation on the rig, mechanical alignment and electrical continuity were rechecked to ensure repeatability. The tests were then carried out at room temperature under continuous unipolar excitation for 180 min. After each run, the bearing was removed, fully disassembled, cleaned with isopropanol to remove residual grease, and air-dried before surface characterization.

### 2.4. Excitation Parameters and Repeatability

The excitation waveform consisted of a unipolar rectangular signal with a fixed frequency of 20 kHz and a current limit of 3 A. Pulse width and duty cycle were maintained constant for both polarity conditions to guarantee equivalent electrical energy input per cycle, with polarity serving as the sole experimental variable. All other parameters were held unchanged. The selected current limit of 3 A was determined based on prior bipolar experiments [19], where measurable EDM-induced damage first emerged at approximately 2.02 A and became clearly pronounced at 3 A. Earlier current–voltage response analysis indicated that increasing current intensity elevated shaft voltage amplitude and discharge frequency. To ensure reproducible and sufficiently observable surface modification, the same 3 A setting was adopted in the present investigation, with the corresponding power supply configuration illustrated in Figure 2. Each polarity condition was repeated in three independent tests to assess repeatability and reduce experimental variability.

### 2.5. SEM and XPS Analysis

After the tests, the dissembled bearings were cut using an electric wiring cutting machine to obtain coupon samples (3 mm × 10 mm × 5 mm) from the outer and inner rings for SEM and XPS analyses. The SEM used is the FEI Quanta 200 Environmental Scanning Electron Microscope (ESEM) (FEI company, Hillsboro, OR, USA) with an EDAX Octane Plus SDD detector (AMETEK, Inc., Mahwah, NJ, USA) and TEAM software. The XPS used is a Kratos AXIS Supra spectrometer. SEM operated at 20 kV was used to analyze the discolored band area on the outer raceway and the inner raceway after Experiment 1 and the discolored areas on the inner raceway and the outer raceway after Experiment 2. XPS with a monochromatic Al Kα source (15 mA, 15 kV, 225 W) was used to characterize the surface status of a discolored area on the raceways. All spectra were collected using an analysis area of approximately 300 μm × 700 μm.

## 3. Results

### 3.1. Visual and Electrical Characterization of Directional Damage Behavior

**Figure 3:**
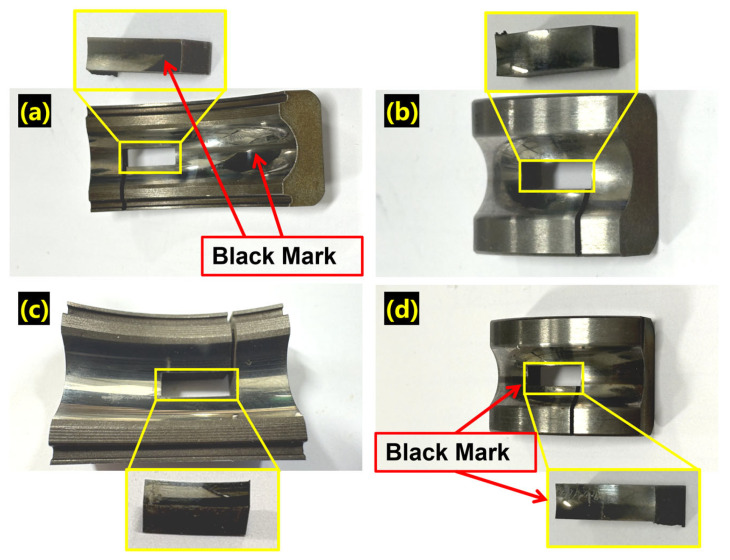
Representative optical images of the raceway surfaces after testing: (**a**) Experiment 1 outer ring, (**b**) Experiment 1 inner ring, (**c**) Experiment 2 outer ring, and (**d**) Experiment 2 inner ring.

Surface damage changed with the excitation polarity (Figure 3). The main observations are summarized below. Under the unipolar-positive condition (Experiment 1, Figure 3a,b), current flowed from the outer ring to the inner ring. A continuous circumferential dark band was observed on the outer raceway. The affected region showed darkened tracks and thermally affected areas within a limited contact path. The inner raceway showed little visible color change.

When the polarity was reversed and current flowed from the inner ring to the outer ring (unipolar-negative, Experiment 2, Figure 3c,d), the visible damaged region shifted to the inner raceway. In this case, the damage appeared mainly as a lighter local discoloration rather than as a continuous dark band. No clear color change was observed on the outer ring. These observations show that the visible damaged region changed with current direction. The two cases also showed different damage morphologies. Under the present test conditions, the outer-ring anode case produced a broader circumferential band, while the inner-ring anode case produced a more localized damaged region. This difference is also consistent with the shaft-voltage results in Figure 2. In the unipolar-negative configuration (Experiment 2, with the inner ring as the current entry side), the positive peak shaft voltage was about 3 V. In the unipolar-positive configuration (Experiment 1, with the outer ring as the current entry side), the peak shaft voltage was about 2 V. The higher voltage in Experiment 2 suggests that discharge initiation required a higher breakdown threshold in that configuration. Under the present conditions, this is consistent with less frequent discharge events and more localized visible damage on the inner raceway. By comparison, the lower voltage in Experiment 1 is consistent with more frequent discharge initiation and a broader damaged band on the outer raceway.

A similar relation between shaft voltage level and electro-erosion severity has been reported in electric vehicle (EV) motor bench experiments [10]. The present results also suggest that lubrication state and contact geometry may contribute to the polarity-dependent behavior. Variations in grease film thickness can change the local capacitive impedance of the rolling contact, which may affect the charging and discharging behavior of the lubricant film [21]. Under the present conditions, the observed asymmetry was likely attributed to combined effects from electrical field distribution, discharge threshold, and contact conditions at the inner and outer raceways.

### 3.2. Microstructural Evidence of Anode-Dominated Degradation

**Figure 4:**
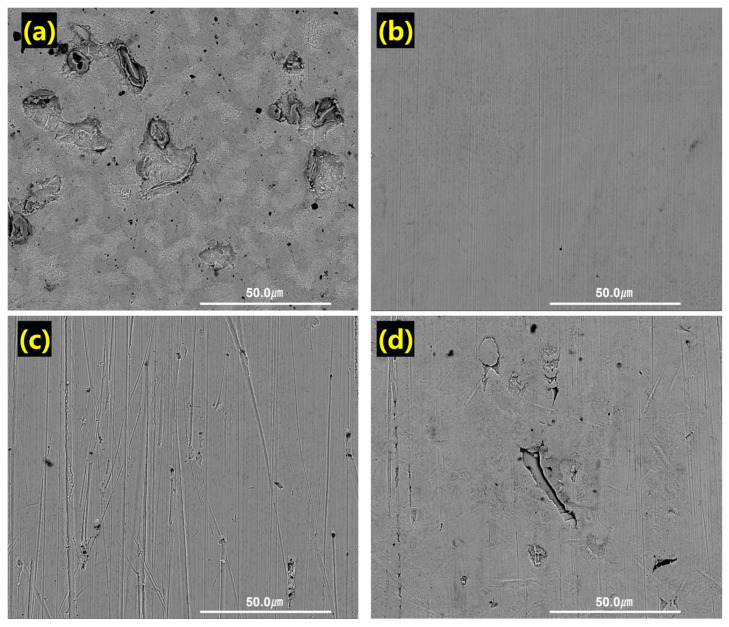
Representative SEM images of the raceway surfaces after testing: (**a**) Experiment 1 outer ring, (**b**) Experiment 1 inner ring, (**c**) Experiment 2 outer ring, and (**d**) Experiment 2 inner ring.

SEM images showed clear differences between the two polarity conditions (Figure 4). In both tests, the raceway connected as the anode showed more discharge-related surface features than the corresponding cathode-side raceway. In the unipolar-positive configuration (Experiment 1), the outer raceway acted as the anode (Figure 4a). This surface showed dense crater formation, local melting features, and ridge-like discharge marks within a narrow circumferential region.

The crater diameters ranged from about 2 μm to 22 μm, and many craters appeared in clusters. By comparison, the inner raceway in the same test (Figure 4b), which acted as the cathode, showed only sparse and isolated marks with smaller crater sizes and no continuous damage band.

When the polarity was reversed (Experiment 2), the damage location changed accordingly. The inner raceway, which acted as the anode in this case (Figure 4d), showed crater formation and mild melting features. The damage was still visible, but both crater density and overall severity were lower than those observed on the outer-ring anode in Experiment 1. The outer raceway in Experiment 2 (Figure 4c) remained largely intact, except for minor surface irregularities. These SEM observations show that the main damaged region was located on the anode-side raceway in both tests. The comparison between Experiment 1 and Experiment 2 also suggests that raceway geometry, lubrication state, and discharge conditions may affect the local severity of surface damage. Under the present test conditions, the outer-ring anode case showed broader and denser surface damage, whereas the inner-ring anode case showed more localized damage.

### 3.3. Mechanical Contact Stability and Discharge Localization

The mechanical configuration of the bearing setup introduced a distinct asymmetry in discharge localization. Under unipolar-positive excitation, the outer ring—connected to the positive terminal—remained stationary, whereas the inner ring rotated with the shaft. As the rolling elements moved along the raceway, their electrical contact with the outer ring occurred repeatedly along the same circumferential path rather than at a single fixed point. This led to the concentration of discharge activity within a narrow annular region on the outer raceway, where localized heating and cumulative material removal were most pronounced. In contrast, under unipolar-negative excitation, the inner ring served as the positive terminal and rotated, causing discharge spreading along the inner raceway surface. According to Hertzian contact theory, the contact area A between a rolling element and the raceway is defined as

(1) $$a={\left(\frac{3Fr^{\prime }}{4E^{\prime }}\right)}^{\frac{1}{3}}$$

(2) $$A_{cnt}=\pi a^{2}$$

where a is the Hertzian contact radius; F is the normal load; $r^{\prime }$ is the reduced radius of curvature; and E′ is the reduced elastic modulus. $A_{cnt}$ denotes the nominal Hertzian contact area.

Although the nominal contact areas were similar for inner and outer raceways, subtle differences in curvature and relative motion resulted in variations in the actual discharge area. According to the properties for each part of the bearing listed in Table 1, the calculated contact area between the ball and outer raceway was $A_{out}=0.00295\;{mm}^{2}$, while that for the inner raceway was $A_{in}=0.00281\;{mm}^{2}$. The corresponding current densities under identical current (Idis = 3 A) for 10 balls are given by

(3) $$J=\frac{I_{dis}}{A_{cnt}}$$

where J is the current density and $I_{dis}$ is the current.

This yields

$$J_{out}=1.02\times 10^{8}\;A/m^{2}$$

$$J_{in}=1.07\times 10^{8}\;A/m^{2}$$

These results imply that the inner ring (as the anode in Experiment 2) experiences a higher theoretical current density than the outer ring in Experiment 1. However, surface analysis indicates that the outer ring in Experiment 1 suffered more severe damage, with more pronounced arc-erosion features. This suggests that the EDM-type damage would be more relevant to the difference in the voltage than in current density mentioned above.

Additional factors, including discharge localization and discharge frequency, should be considered. In the unipolar-positive configuration (Experiment 1), the outer ring remained stationary and was subjected to rolling and sliding contact with the rolling elements, which resulted in localized thermal accumulation and crater overlap, amplifying surface degradation.

Therefore, the difference in degradation may be attributed to variations in electrical contact capacitance (C) and equivalent impedance (Z) rather than to average current density alone. A larger effective capacitance and lower impedance on the stationary outer raceway likely enable faster charge accumulation and more frequent dielectric breakdown events. Conversely, the rotating inner ring presents a slightly higher impedance and less stable charge buildup, leading to fewer discharge occurrences. These electrical parameters, together with discharge localization and motion-induced dispersion, provide a more consistent explanation for why the outer raceway in Experiment 1 exhibited more pronounced arc-erosion damage than the inner raceway in Experiment 2 as evidenced in the following sections.

### 3.4. Grease Distribution and Dielectric Barrier Effects

At low rotational speeds, grease distribution within the bearing becomes uneven due to gravitational effects and limited centrifugal force [19]. This behavior leads to differences in dielectric film thickness between the inner and outer raceways [21]. Specifically, grease tends to accumulate preferentially along the inner raceway [22], resulting in a thicker insulating layer $d_{in}$, while the outer raceway remains more exposed and forms a thinner dielectric layer $d_{out}$. Given the capacitance expression, it follows that

(4) $$C=\epsilon \cdot \frac{A_{cnt}}{d}$$

where C is the electrical capacitance across the lubricant film; ϵ is the dielectric permittivity of the lubricant; $A_{cnt}$ is the effective contact area between the rolling element and the raceway; and d is the local dielectric film thickness. Assuming a constant dielectric constant ε, given that the same lubricant was used in both experiments, it follows that

$$C_{in}<C_{out}\cdot \sin\mathrm{ce}\;d_{in}>d_{out},\;A_{in}<A_{out}$$

The subscripts “in” and “out” denote the inner and outer raceways, respectively. Thus, $C_{in}$ and $C_{out}$ represent the capacitances at the inner and outer contacts; $d_{in}$ and $d_{out}$ are the corresponding lubricant film thicknesses; and $A_{in}$ and $A_{out}$ are the respective Hertzian contact areas.

This relationship indicates that the outer raceway can more readily accumulate charge due to its larger effective surface area and its stationary position within the current path. Because the inner raceway carries a thicker lubricant film and a smaller effective contact area ${(d}_{in}>d_{out},\;A_{in}<A_{out}),$ its capacitance is lower $\left(C_{in}<C_{out}\right)$; the inner contact therefore stores less charge per unit voltage and requires a higher voltage to reach dielectric breakdown. Because the outer ring remains electrically static while the inner ring rotates, charge dissipation through motion or micro-sliding is minimized, allowing localized charge buildup along the contact track [22]. Consequently, the outer raceway reaches the threshold for dielectric breakdown more easily, particularly under unipolar-positive excitation, where it functions as the anode. Although calculated current density J on the inner raceway is slightly higher due to its smaller contact area, the thinner dielectric barrier on the outer ring facilitates earlier and more frequent discharge events. This explains why, in Experiment 1, the outer ring in unipolar-positive mode exhibited more severe damage than the inner ring in unipolar-negative mode—even though both served as anodes in their respective configurations.

Therefore, the combination of a thinner dielectric layer and higher local capacitance for breakdown on the outer raceway provides an explanation for the observed polarity-driven asymmetry in damage severity.

### 3.5. Shaft Voltage Differences and Discharge Readiness

The shaft voltage discrepancy observed between the unipolar-positive (2 V) and unipolar-negative (3 V) conditions can be explained through qualitative analysis using a resistor capacitor (RC) circuit impedance model. The capacitive impedance is defined as

(5) $$Z_{C}=\frac{1}{2\pi fC}$$

(6) $$Z_{eq}={\left(\frac{1}{R}+\frac{1}{Z_{C}}\right)}^{-1}$$

where $Z_{C}$ is the capacitive impedance of the lubricant film, f is the excitation frequency, and C is the contact capacitance. $Z_{eq}$ denotes the equivalent impedance of the RC branch. R is the effective contact resistance associated with the conductive discharge path, and $Z_{C}$ is the capacitive impedance defined in Equation (5).

Given a constant excitation frequency of 20 kHz, and measured contact resistances of $R_{out}=0.9\;\Omega$ for the outer ring and $R_{in}=0.8\;\Omega$ for the inner ring, the difference in effective capacitance becomes the primary factor governing the total impedance. Based on lubrication behavior, the inner ring is coated with a thicker grease layer, leading to a lower capacitance $C_{in}<C_{out}$, and hence

$$Z_{C,in}>Z_{C,out}$$

Substituting into the equation, it follows that

(7) $$Z_{eq,out}={\left(\frac{1}{0.9}+\frac{1}{Z_{C,out}}\right)}^{-1},\;Z_{eq,in}={\left(\frac{1}{0.8}+\frac{1}{Z_{C,in}}\right)}^{-1}\;$$

Since $Z_{C,in}>Z_{C,out},$ the total equivalent impedance $Z_{eq,in}>Z_{eq,out}$. This result aligns with oscilloscope measurements showing that a higher shaft voltage (3 V) was required to initiate discharge in the unipolar-negative mode (inner ring as anode), while discharges occurred more readily at lower voltage (2 V) in the unipolar-positive mode (outer ring as anode). Thus, the variation in local capacitance and impedance can explain the polarity-dependence of electrical discharge.

### 3.6. Polarity-Induced Asymmetry: Anode-Side Raceway as the Primary Damage Zone

Across both tests, the raceway connected as the positive terminal showed more visible discharge-related damage than the raceway connected as the negative terminal. In Experiment 1, the outer raceway acted as the anode and showed dense craters, melting marks, and a broader damaged band. In Experiment 2, after polarity reversal, the main damaged region shifted to the inner raceway, while the outer raceway showed only limited marks. These observations indicate that, under the present conditions, the main damaged region was located on the anode-side raceway. The results therefore suggest that electrical polarity, together with local contact conditions, affects where EDM-type damage is concentrated in the bearing.

### 3.7. EDS and XPS Surface Chemical Analysis for Damaged Region

**Figure 5:**
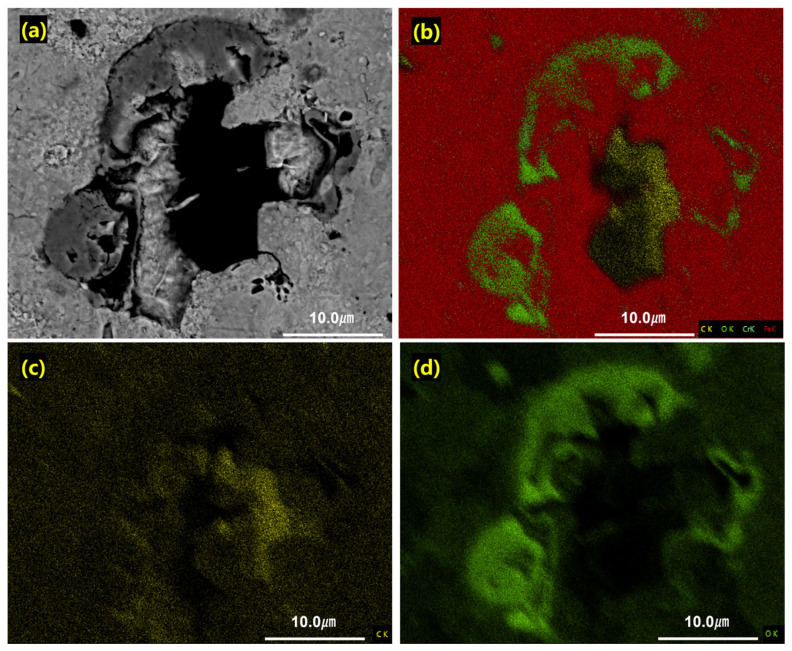
Representative SEM and EDS results from a discharge-affected region: (**a**) SEM image of the damaged area, (**b**) element mapping, (**c**) carbon mapping, and (**d**) oxygen mapping.

The SEM and EDS results shown in Figure 5 highlight the localized nature of electrical discharge-induced degradation observed at high magnification. Table 2 summarizes the elemental composition in terms of weight percentage (wt.%) for both the damaged and undamaged areas. The SEM images reveal that the black-marked regions (damage region) on the bearing surfaces contain distinct crater-like features, whereas the adjacent bright regions remain largely smooth and undisturbed.

EDS elemental mapping of these cratered zones showed elevated oxygen (O) concentrations around the discharge-affected region. Carbon-related signals were also observed in the mapped area, but the present EDS results do not allow a definite interpretation of their origin. Table 2 indicates a higher O content in the black damaged area than in the undamaged areas (9.8 wt.% vs. 5.3 wt.%). As observed in Figure 3a, visually apparent black bands formed on the raceways even when the surface morphology remained less obvious under low magnification. The location and intensity of these markings may be influenced by grease film thickness, dielectric strength, bearing geometry, and rotational conditions.

**Figure 6:**
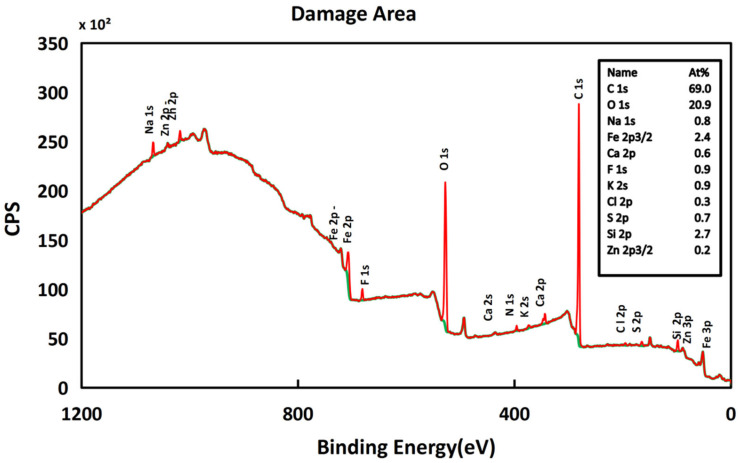
XPS survey spectrum collected from the discharge-affected region.

**Figure 7:**
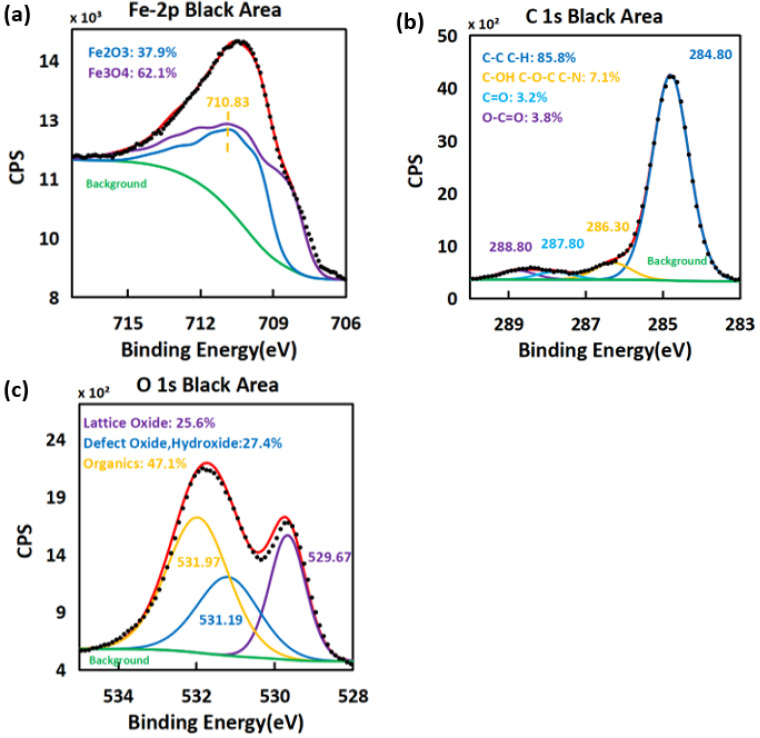
High-resolution (**a**) Fe 2p, (**b**) C 1s, (**c**) O 1s XPS spectra of the damaged black region.

These darkened regions should therefore be interpreted mainly as discharge-affected surface zones rather than as areas of conventional mechanical wear. To further examine the surface chemistry of these zones, XPS was carried out on the region identified by optical and SEM inspection as electrically affected. The XPS survey spectrum of the damaged region (Figure 6) showed strong carbon and oxygen signals together with a reduced Fe signal. The measured surface composition was approximately 69 at.% C, 21 at.% O, and only about 2–3 at.% Fe. This result indicates that the steel substrate was covered by a surface film formed during the test. The Fe 2p spectrum in Figure 7 was dominated by oxidized iron species, with the main contribution located near 710.83 eV and no clear metallic Fe peak near 706.5–707 eV under the present measurement conditions. This observation is consistent with the presence of an oxidized surface layer on the damaged region.

A comparison with established non-damage XPS profiles from the literature further reinforces this interpretation [11]. For instance, the non-damaged bearing steel typically exhibits significantly higher Fe intensities (often exceeding 20 at.%) along with lower carbon content in the range of less than 30 at.% and some contributions from oxidized species [11]. In contrast, the damaged region in the present work shows an extensive carbon layer and near-complete suppression of metallic Fe around the peripheral edges of the EDM-induced craters. Such features were not observed on the intact raceway surfaces. These differences confirm that the analyzed region has undergone substantial physicochemical transformation attributable to electrical stress.

High-resolution Fe 2p spectra (Figure 7a) provide additional evidence of discharge-induced modification. The Fe 2p_3/2_ peak consists entirely of oxidized iron species, dominated by Fe_3_O_4_-like mixed-valence oxides and Fe_2_O_3_/FeOOH-type Fe^3+^ oxides (near 710.83 eV), with no detectable metallic Fe^0^ signal near 706.5–707 eV. Non-damaged steel surfaces generally retain a measurable metallic Fe component or exhibit a thin native oxide layer [11]. The absence of Fe^0^ in the present analysis therefore indicates extensive oxidation consistent with localized arc heating and repetitive discharge events. The C 1s spectrum in the black damaged area shows a strong peak at 284.80 eV, which corresponds to C–C/C–H bonding (Figure 7b). Several weaker components are also fitted in this spectrum, including C–OH/C–O–C/C–N, C=O, and O–C=O. These peaks indicate that the carbon signal is not only from hydrocarbon-type species but also includes oxidized carbon species. In the O 1s spectrum (Figure 7c), the fitted peaks are related to lattice oxide, defect oxide, hydroxide, and organic oxygen. The oxygen signals were associated with the oxidized Fe species discussed in the Fe 2p spectrum. Based on the Fe 2p, C 1s, and O 1s spectra, the black damaged region contains both iron oxides and carbon-rich residues. This result suggests that local discharge heating can change the raceway surface through oxidation and lubricant decomposition.

## 4. Conclusions

This work demonstrated that current polarity affected where electrical damage appeared on the bearing raceway under the present test conditions. When the outer ring acted as the anode (i.e., current flowed from housing to shaft), the EDM damage was more extensive on the outer raceway. When the polarity was reversed (i.e., current flowed from shaft to housing), the EDM damage was shifted to the inner raceway. SEM results showed evidence of differences in erosion crater density and melting features between the two cases. XPS analysis confirmed surface oxidation and chemically altered raceway surfaces in the damaged areas. Under the present low-rotating-speed and high-frequency unipolar excitation conditions, current direction was indeed found to be an important factor in the spatial distribution of EDM-type damage. This point should be considered when evaluating shaft-current-related bearing damage and when designing mitigation measures.

## Figures and Tables

**Table 1:** Properties for each part of the bearing.

	E (GPa)	m (g)	Poisson’s Ratio	r (mm)	r’ (mm)	A_cnt_ (mm^2^)
Ball	210	5.10	0.3	6.70	~	~
Outer Ring	210	213.19	0.3	8.72	3.78	0.00295
Inner Ring	210	~	0.3	7.12	3.45	0.00281

**Table 2:** Element weight percentage (wt.%) of damaged and undamaged areas.

Element	Damaged Region (wt.%)	Undamaged Region (wt.%)
Fe	72.27	83.74
C	NA	NA
Cr	1.02	1.51
O	9.8	5.3
Others	Balanced	Balanced

## Data Availability

The original contributions presented in this study are included in the article. Further inquiries can be directed to the corresponding author.

## Abbreviations

The following abbreviations are used in this manuscript:

DC	direct current
SEM	scanning electron microscopy
XPS	X-ray photoelectron spectroscopy
EDM	electrical discharge machining
WLI	white light interferometry
FIB	focused ion beam
EDS	energy dispersive spectrometer
EV	electric vehicle
RC	resistor capacitor
C	carbon
Cr	chromium
O	oxygen
wt.	weight
Fe	iron
rpm	revolution(s) per minute
a	Hertzian contact radius
F	normal load
$r^{\prime }$	reduced radius of curvature
E′	reduced elastic modulus
$A_{cnt}$	Hertzian contact area
J	current density
$I_{dis}$	discharge current
C	electrical contact capacitance
Z	equivalent impedance
ϵ	dielectric permittivity of the lubricant
$A_{cnt}$	effective contact area between the rolling element and the raceway
$C_{in}$	capacitance at the inner contacts
$C_{out}$	capacitance at the outer contacts
$d_{in}d_{in}$	corresponding inner raceway lubricant film thicknesses
$d_{out}d_{in}$	corresponding outer raceway lubricant film thicknesses
$Z_{C}$	capacitive impedance of the lubricant film
$Z_{eq}$	equivalent impedance of the RC branch
R	effective contact resistance associated with the conductive discharge path
